# Low-dose corticosteroid combined with immunoglobulin reverses deterioration in severe cases with COVID-19

**DOI:** 10.1038/s41392-020-00407-0

**Published:** 2020-11-24

**Authors:** Zhi-Guo Zhou, Di-Xuan Jiang, Shu-Min Xie, Jing Zhang, Fang Zheng, Hong Peng, Xuan Chen, Ji-Yang Liu, Lei Zhang

**Affiliations:** 1 Department of Respiratory Medicine, The First Hospital of Changsha, 410000 Changsha, Hunan China; 2 The Xiangya Hospital, Central South University, 410000 Changsha, Hunan China; 3 Hong Kong Polytechnic University, 999077 Hong Kong, China; 4 Department of Infectious Diseases, The First Hospital of Changsha, 410000 Changsha, Hunan China; 5 Department of Respiratory Medicine, The Second Xiangya Hospital, Central South University, 410000 Changsha, Hunan China; 6 Tianjin Medical University, 300000 Tianjin, China; 7 The First Hospital of Changsha, 410000 Changsha, Hunan China; 8 Tianjin Medical University Cancer Institute and Hospital, 300000 Tianjin, China

**Keywords:** Infectious diseases, Respiratory tract diseases

**Dear Editor**,

Current coronavirus epidemic swept all over the world, infected over 37 million people and resulted in 1 million deaths.^1^ With a mortality rate in critically ill patients up to 61.5% and a limited effective treatment options, it is of top priority to explore treatments to prevent the clinical deterioration from severe cases to critically ill patients.^1^ Here, we share our detailed experience with 239 cases of COVID-19, including 40 severe cases. Through early and innovative treatment with low-dose corticosteroid combined with immunoglobulin, we achieved good clinical outcomes in overall mortality rate (0.84%) and morality rate of severe cases (5%).

We studied 239 COVID-2019 patients in The North Yard of The First Hospital of Changsha (Changsha Public Health Center) from January 17th to March 14th 2020. The epidemiological, clinical, laboratory, radiographic, and Acute Physiology Chronic Health Evaluation II (APACHE II) scores for all patients were collected (Supplementary Table S1). Among the 40 severe cases, the proportion of patients with comorbidity reached 50%, while for the nonsevere patients, the percentage was only 17.1% (*P* < 0.05). The severe patients were generally older than then nonsevere patients (*P* < 0.05), and had a higher exposure to Wuhan (*P* < 0.05) and to Wuhan citizens (*P* < 0.05).

All 40 severe COVID-19 cases were treated with a combination of low-dose corticosteroid and immunoglobulin regimen. In our study, the initial dose of methylprednisolone was 40 mg/d for six cases, and 80 mg/d for the other 34 severe cases. The duration of methylprednisolone was 4–25 days, with an average of 10.28 ± 4.85 days. A total of 11 cases received a pulse dose of 160 mg/d, (duration for 2–7 days with an average of 4.09 ± 1.70 days, nine of these 11 severe cases went into remission after this treatment). The initial dose of immunoglobulin was 10 g/d for 35 cases, and 20 g/d for the other five cases. The duration of immunoglobulin was 4–26 days, with an average of 10.13 ± 4.50 days. Meanwhile, 10.6% of 199 nonsevere cases who experienced deterioration also received a combination of low-dose corticosteroid combined with immunoglobulin to prevent progression to severe cases. More details about this innovative precise use of corticosteroid and immunoglobulin, including clinical indicators, timing, dosage and duration, were shown in the “Materials and methods” in the supplementary material.

We evaluate the therapeutic effect of the therapy through comparing the clinical parameters throughout the treatment. Our results showed that the 40 severe cases achieved significant improvement with treatment in terms of vital signs, blood work, and the APACHE II scores when compared to the peak values (Fig. 1a–e and Supplementary Table S2). The PaO_2_/FiO_2_ (*P* < 0.05) was significantly improved. The APACHE II score was significantly lower (*P* < 0.05). The patients’ body temperature (*P* < 0.05) significantly decreased to normal level. An essential prognostic index, the lymphocyte count (*P* < 0.05) significantly increased to normal level. In terms of inflammation-related biomarkers, CRP (*P* < 0.05) was improved. The complete blood count, including leukocytes, neutrophils, and platelets increased significantly after treatment (*P* < 0.05). The liver and kidney function tests including albumin, alanine aminotransferase (ALT), aspartate aminotransferase (AST), total bilirubin (TBIL), and creatinine were significantly improved (*P* < 0.05). The myocardial enzymes, creatine kinase (CK), and lactate dehydrogenase (LDH) was significantly lower than the peak value (*P* < 0.05). In terms of oxygenation index, SPO_2_, PaCO_2_, and lactic acid were all significantly improved (*P* < 0.05) (Supplementary Table S2).

**Fig. 1:**
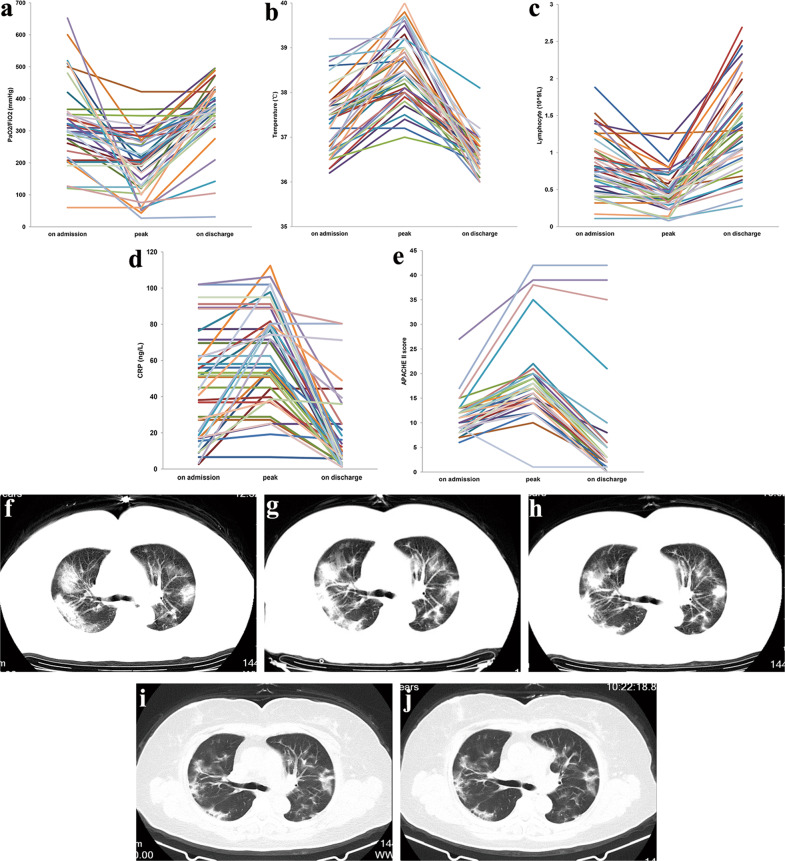
**a**–**e** Trendgraph of important clinical variables of 40 severe cases respecting to the low-dose corticosteroid combined with immunoglobulin. **a** PaO_2_/FiO_2_, **b** temperature, **c** lymphocyte, **d** CRP, **e** APACHE II score. **f**–**j** A dynamic series of pulmonary CT imaging manifestation of a severe case. Multiple patchy, ground glass, and infiltrating shadows in bilateral lungs on hospital admission. During the treatment, the follow-up CT changes presented an improvement type (the imaging lesions continually absorbed). **f** The day before treatment with low-dose corticosteroid combined with immunoglobulin, **g** the 4th day of treatment, **h** the 9th day of treatment, **i** the 13th day of treatment, **j** the 3rd day after treatment

The common early pulmonary CT findings of COVID-19 infected patients showed multiple small, patchy infiltrates and interstitial changes in bilateral lungs; this progressed to bigger infiltrates with ground glass appearance in some cases. Pleural effusion was rare. A typical pulmonary CT series of severe case is displayed in Fig. 1f–j. Re-examination of the pulmonary CT of the 40 severe cases showed that the lung lesions improved in 36 cases, were stable in two cases, and deteriorated in only two cases. As of March 14th, a total of 237 cases in this study were discharged. Only two deaths occurred in our study; the probable cause of one death was cardiac failure secondary to acute myocardial injury (Supplementary Table S3).

As a double-edged sword in the treatment of viral pneumonias, corticosteroids must be administered properly and precisely.^2^ Instead of conventional strategy of administrating corticosteroid in the final stages of a viral pneumonia, we believe that early low-dose corticosteroid therapy should be under consideration when clinical data indicates the progression of COVID-19. The relatively mild inflammatory response in the early stage of COVID-19 pneumonia allows low-dose of corticosteroid to control the progression of inflammation.^2^ In our study, this therapy successfully reversed deterioration in most severe cases as shown by the significant improvement in multiple clinical indicators, including respiratory and oxygenation indicators, laboratory indicators, pulmonary imaging, and, most importantly, by the overall decreased mortality. Zheng et al. also reported that low-dose methylprednisolone reduced clinical manifestations of COVID-19 in severely ill patients and lowered the risk of death for those who developed ARDS.^3^ According to our experience, the administration dose, timing, and duration should be determined according to clinical indicators including SPO_2_, body temperature, lymphocyte count, and progression in pulmonary CT.

Besides, we strongly recommend the combined use of immunoglobulin with corticosteroid, which could strengthen the patients’ immune response to win us a relatively safer and longer timeframe for corticosteroid therapy. Immunoglobulin plays an assistant role through controlling the systemic inflammation, reducing lung viral load, and avoiding complications including secondary infection caused by corticosteroid immunosuppression.^4^ Recent retrospective cohort study of 46 severe COVID-19 patients also showed promising results from the combination therapy, with great improvement in oxygen saturation, hospitalization, ICU time, and CT.^5^

In this research, we formally proposed for the first time that combination of low-dose corticosteroid and immunoglobulin can be used to treat severe cases with COVID-19, which not only can reduce the effect of inflammatory storm caused by COVID-19 infection through low-dose corticosteroid, but also can improve the passive immunity and reduce the risk of secondary infections through immunoglobulin, thereby significantly reducing the overall mortality rate and morality rate of severe cases. Our research is of great significance in establishing the important position of low-dose corticosteroid in the treatment of COVID-19, and proposing an innovative combination treatment strategy of low-dose corticosteroid and immunoglobulin. We call for relevant clinical trials to further confirm the effectiveness of this treatment in COVID-19 severe cases.

## Supplementary information

Supplementary materials

## Data Availability

All data generated during this study are included in this published article and its supplementary information files.
