# Supplementary material for: Low-dose corticosteroid combined with immunoglobulin reverses deterioration in severe cases with COVID-19
Source: Signal Transduct Target Ther. 2020 Nov 24;5:276. doi: 10.1038/s41392-020-00407-0 (PMC7683873; doi:10.1038/s41392-020-00407-0)
Supplement: Supplementary file 1 — Supplementary materials [file 41392_2020_407_MOESM1_ESM.docx]

Supplementary Materials for

**Low-dose corticosteroid combined with immunoglobulin reverses deterioration in severe cases with COVID-19**

Zhi-Guo Zhou M.D.^1*^, Di-Xuan Jiang M.D.^1*^, Shu-Min Xie M.D.^2*^, Jing Zhang M.D.^3*^, Fang Zheng M.D.^4^, Hong Peng M.D.^5^, Xuan Chen M.D.^6^, Ji-Yang Liu M.D.^7#^, Lei Zhang M.D.^8#^

Correspondence to: [raymd728@qq.com](mailto:raymd728@qq.com), or [Chan1498@163.com](mailto:Chan1498@163.com)

**This PDF file includes:**

Materials and Methods

Table S1. Demographic and clinical characteristics of 40 severe and 199 non-severe cases with COVID-19

Table S2. Comparison between clinical variables of 40 severe cases respect to the combination of low-dose corticosteroid and immunoglobulin

Table S3. Clinical outcomes of 40 severe cases with COVID-19

**Materials and Methods**

*Participants*

The North Yard of The First Hospital of Changsha (Changsha Public Health Center) is the referral center for the COVID-2019 patients in Changsha, Hunan Province. From January 17^th^ to March 14^th^ 2020, 239 COVID-19 patients including 40 severe cases were treated there. Throat swab specimens were collected at admission and confirmed COVID-19 for all patients. According to the guideline published by National Health Council of China, the severe cases were diagnosed based on one of the following criteria: 1. respiratory distress with respiratory rate (RR) ≥ 30 times per min; 2. SPO_2_≤93%; 3. PaO_2_/FiO_2_≤300mmHg. In our study, the non-severe cases included the mild and moderate cases. Mild cases were defined as individuals who had mild clinical symptoms, such as dry cough, low-grade fever or body aches, and no pneumonia manifestation on imaging. Moderate cases were defined as individuals who have obvious signs such as fever, respiratory tract symptoms, and pneumonia manifestation on imaging, with SPO_2_>93%. Critically ill cases were defined as patient with respiratory failure, septic shock, and/or multiple organ dysfunction or failure. This study was approved by the First Hospital of Changsha Ethics Committee.

*Procedures*

For all patients the following information was collected: epidemiological, clinical, laboratory, radiographic (chest X-ray or pulmonary computed tomography, CT) and Acute Physiology Chronic Health Evaluation Ⅱ (APACHE Ⅱ) scores (Table 1). The laboratory variables included complete blood count, electrolytes, coagulation studies, renal and liver function, myocardial enzymes, C-reactive protein (CRP), erythrocyte sedimentation rate (ESR) and blood gas analysis. Pre- and post-treatment pulmonary CT were compared. Pulmonary radiographic abnormalities were classified as improved, stable or deteriorating: 1. Improved: pulmonary lesions reduced by more than 25%; 2. Stable: pulmonary lesions reduced or increased by less than 25%; 3. Deteriorating: pulmonary lesions increased by more than 25%.

*Treatment protocol*

During hospitalization, a systematic treatment protocol was carried out, including oxygen therapy (low-flow nasal cannula, high-flow nasal cannula, non-invasive mechanical ventilation, or invasive mechanical ventilation), antiviral treatment, antibiotic treatment, antifungal treatment, ECMO, continuous renal replacement therapy (CRRT), combination of low-dose corticosteroid and immunoglobulin.

All 40 severe COVID-19 cases were treated with a combination of low-dose corticosteroid and immunoglobulin regimen. The corticosteroid usage followed the principle of stepwise increment and decrement. The standard corticosteroid regimen was 1-2 mg/kg (40-80mg) methylprednisolone daily for 7-14 days; and the immunoglobulin regimen was synchronized with corticosteroid, 10-20g daily for 7-14 days. The immunoglobulin used was the human immunoglobulin (pH4) for intravenous injection, 2.5g/bottle, 5% concentration, 50ml, Lanzhou Lansheng Blood Products Co., Ltd., China. The initial dose of immunoglobulin was 10g/d, and when patients’ condition continued to deteriorate, the dose was doubled to 20g/d synchronized with corticosteroid increment.

How patients benefit from this therapy is determined by careful and precise use of corticosteroids, influenced by key factors such as clinical indicators, timing, dosage, and duration. We propose an early, low dose, short-term pulse, and step reduction corticosteroid therapy in our protocol. According to our experience, for severe patients with SPO_2_≤93% and no other clinical abnormality, the initial dose should be 40mg/d methylprednisolone; while for patients with SPO_2_ ≤ 93% accompanied by another clinical abnormality including high fever (T>39℃), lymphopenia (lymphocyte count < 0.8*10^9^/L persistently), 80mg/d methylprednisolone is recommended as the initial dose. For patients with significant deterioration including a significant decrease in PaO_2_/FiO_2_, an obvious deterioration in pulmonary CT (>50% lesions progression within 24 hours), a persistently decrease in lymphocyte count (< 0.8*10^9^/L), or a ongoing high fever (T>39℃ for more than 48-72 hours), the dose of methylprednisolone should be doubled, with a maximum dose ≤ 160mg/d. In our study, the pulse dose of 160mg/d did not generally exceed 5 days. Once the patients’ condition was stable (a normal temperature, an increased PaO_2_/FiO_2_, and no progression in pulmonary CT) for 48-72 hours, the dose of methylprednisolone decreased gradually to 120mg, 80mg, and 40mg daily. Once the patient condition stabilized on 40mg/d of methylprednisolone, the withdrawal of methylprednisolone should be considered.

*Evaluation of therapeutic effect*

Improvement in clinical parameters, including vital signs, laboratory markers, pulmonary CT, as well as the APACHE Ⅱ score were used to evaluate the therapeutic effect. To account for individual patient illness severity and recovery, peak values, corresponding with the patient’s worst clinical status, were noted. According to the guideline from National Health Council of China, discharge and release from quarantine criteria had to meet all the following criteria: 1. afebrile for more than 3 days; 2. respiratory symptoms significantly relieved; 3. abnormal imaging findings substantially improved; 4. negative COVID-19 nucleic acid test for two consecutive respiratory samples (sampling interval ≥ 1 day).

*Statistical analysis*

SPSS16.0 software was used for statistical analysis. P <0.05 was considered statistically significant. Numeration data were described by number (%), and measurement data were described by mean ± standard deviation. The paired sample t test was used to evaluate the changes of clinical indexes on admission and discharge, as well as the peak value. Independent sample t test was adopted to compare the difference of age and course between severe and non-severe cases. Pearson Chi-Square test was used for numeration data analysis.

Table S1 Demographic and clinical characteristics of 40 severe and 199 non-severe cases with COVID-19

| Characteristics | Severe cases (n=40) | Non-severe cases (n=199) | P^*^ |
| --- | --- | --- | --- |
| Age (range) | 55.28±13.89 year-old  (25~78) | 43.76±16.44 year-old  (1~82) | 0.000 |
| Gender |  |  | 0.507 |
| Male | 22 (55.0%) | 98 (49.2%) |  |
| Female | 18 (45.0%) | 101 (50.8%) |  |
| Days between onset and hospital admission (range) | 7±3.74 days  (1~15) | 6·08±4.58 days  (1~30) | 0.232 |
| Medical staff | / | 3 (1.5%) | / |
| Positive result of 2019-nCoV nucleic acid | 40 (100.0%) | 199 (100.0%) | / |
| Comorbidities | 20 (50.0%) | 34 (17.1%) | 0.000 |
| Hypertension | 14 (35.0%) | 21 (10.6%) |  |
| Cerebrovascular disease | 1 (2.5%) | 5 (2.5%) |  |
| Diabetes | 4 (10.0%) | 10 (5.0%) |  |
| Coronary heart disease | 5 (12.5%) | 7 (3.5%) |  |
| COPD | 2 (5.0%) | 2 (1.0%) |  |
| Hepatic cirrhosis | 1 (2.5%) | / |  |
| Arrhythmia | / | 2 (1.0%) |  |
| Gastric ulcer | / | 3 (1.5%) |  |
| Pericarditis | / | 1 (0.5%) |  |
| Symptoms |  |  | / |
| Fever | 35 (87.5%) | 153 (76.9%) |  |
| Dry cough | 24 (60.0%) | 84 (42.2%) |  |
| Expectoration | 10 (25.0%) | 41 (20.6%) |  |
| Dyspnea | 17 (42.5%) | 7 (3.5%) |  |
| Fatigue | 15 (37.5%) | 59 (29.6%) |  |
| Nausea and vomiting | 2 (5.0%) | 9 (4.5%) |  |
| Dizziness | 2 (5.0%) | 9 (4.5%) |  |
| Headache | 11 (27.5%) | 10 (5.0%) |  |
| Myalgia | 5 (12.5%) | 18 (9.0%) |  |
| Pharyngalgia | 5 (12.5%) | 23 (11.6%) |  |
| Diarrhea | 7 (17.5%) | 14 (7.0%) |  |
| Abdominal pain | / | 3 (1.5%) |  |
| Anorexia | 17 (42.5%) | 23 (11.6%) |  |
| Chest distress | 3 (7.5%) | 12 (6.0%) |  |
| Thirst | 2 (5.0%) | 1 (0.5%) |  |
| Nasal obstruction and discharge | / | 6 (3.0%) |  |
| Chest pain |  | 7 (3.5%) |  |
| Epidemiology |  |  |  |
| Wuhan exposure | 25 (62.5%) | 88 (44.2%) | 0.035 |
| Wuhan citizen exposure | 28 (70.0%) | 101 (50.8%) | 0.026 |
| Cluster onset | 13 (32.5%) | 99 (49.7%) | 0.046 |
| Treatments |  |  |  |
| Oxygen therapy | 40 (100.0%) | 99 (49.7%) | 0.000 |
| Low-flow nasal cannula | 18 (45.0%) | 99 (49.7%) |  |
| High-flow nasal cannula | 18 (45.0%) | / |  |
| Non-invasive mechanical ventilation | 8 (20.0%) | / |  |
| Invasive mechanical ventilation | 4 (10.0%) | / |  |
| Etiology treatment |  |  | / |
| Antiviral treatment | 40 (100.0%) | 199 (100.0%) |  |
| Antibiotic treatment | 38 (95.0%) | 78 (39.2%) |  |
| Antifungal treatment | 6 (15.0%) | / |  |
| Combination of low-dose corticosteroid and immunoglobulin | 40 (100.0%) | 21(10.6%) | 0.000 |
| Low-dose corticosteroid only | / | 11 (5.5%) | / |
| Immunoglobulin only | / | 3 (1.5%) | / |
| Extracorporeal membrane oxygenation | 3 (7.5%) | / | / |
| Continuous renal replacement therapy | 4 (10.0%) | / | / |

^*^P<0.05 is of statistical significance

Table S2 Comparison between clinical variables of 40 severe cases respect to the combination of low-dose corticosteroid and immunoglobulin

| Variables | On admission | Peak  (highest or lowest) | On discharge | P^*#^ | P^*&^ |
| --- | --- | --- | --- | --- | --- |
| **Signs** |  |  |  |  |  |
| Temperature (℃) | 37.39±0.75 | 38.55±0.73 | 36·57±0·45 | 0.000 | 0.000 |
| Respiratory rate (times per minute) | 20.65±2.82 | 24.70±6.39 | 20.30±1.24 | 0.515 | 0.000 |
| Heart rate (times per minute) | 91.95±15.35 | 108.58±15.26 | 82.88±18.27 | 0.025 | 0.000 |
| SPO_2_ (%) | 95.23±3.28 | 91.53±4.94 | 96.78±1.44 | 0.005 | 0.000 |
| **Laboratory variables** |  |  |  |  |  |
| **Blood Routine** |  |  |  |  |  |
| Leukocyte (10^9^/L, normal range 4.0-10.0) | 4.71±1.97 | 4.21±2.03 | 8.70±3.73 | 0.000 | 0.000 |
| Lymphocyte (10^9^/L, normal range 0.8-4.0) | 0.82±0.40 | 0.49±0.26 | 1.36±0.61 | 0.000 | 0.000 |
| Neutrophil (10^9^/L, normal range 2.0-7.0) | 3.80±2.02 | 12.01±4.67 | 5.62±3.56 | 0.010 | 0.000 |
| Platelet (10^9^/L, normal range 100.0-300.0) | 179.28±82.66 | 150.20±74.66 | 245.12±107.62 | 0.000 | 0.000 |
| **Coagulation function** |  |  |  |  |  |
| Prothrombin time (s, 10.0-15.0) | 12.33±0.84 | 13.51±2.84 | 11.74±2.71 | 0.192 | 0.000 |
| Activated partial thromboplastin time (s, 26.2-46.0) | 32.29±3.38 | 37.23±13.48 | 30.94±8.41 | 0.341 | 0.000 |
| **Blood biochemistry (normal range)** |  |  |  |  |  |
| Sodium (mmol/L, 133.0-149.0) | 134.17±2.87 | 132.03±2.90 | 136.30±2.13 | 0.000 | 0.000 |
| Potassium (mmol/L, 3.5-5.5) | 3.79±0.36 | 5.06±0.72 | 4.25±0.39 | 0.000 | 0.000 |
| Calcium (mmol/L, 2.0-2.6) | 1.46±0.43 | 1.19±0.16 | 1.23±0.18 | 0.002 | 0.000 |
| Albumin (g/L, 35.0-55.0) | 33.72±3.83 | 27.68±2.48 | 35.60±4.62 | 0.069 | 0.000 |
| Alanine aminotransferase (U/L, 0.0-40.0) | 25.96±13.46 | 128.53±233.62 | 41.09±32.50 | 0.003 | 0.018 |
| Aspartate aminotransferase (U/L, 0.0-37.0) | 34.84±15.23 | 155.35±472.63 | 108.00±456.65 | 0.310 | 0·018 |
| Total bilirubin (umol/L, 3.4-20.5) | 12.23±5.35 | 21.96±13.52 | 13.18±13.98 | 0.696 | 0.000 |
| Creatinine (umol/L, 22.0-133.0) | 60.84±43.54 | 96.67±85.77 | 68.59±50.35 | 0.369 | 0.001 |
| Blood urea nitrogen (mmol/L, 2.8-8.2) | 6.08±2.92 | 11.26±7.57 | 7.05±5.23 | 0.271 | 0.000 |
| Creatine kinase (U/L, 10.0-190.0) | 180.42±213.50 | 313.93±519.00 | 91.33±327.66 | 0.083 | 0.001 |
| Creatine kinase-MB (U/L, 0.0-23.0) | 12.87±8.43 | 57.21±203.57 | 15.36±14.10 | 0.235 | 0.184 |
| Lactate dehydrogenase (U/L, 80.0-245.0) | 249.73±84.12 | 462.25±767.27 | 224.98±122.29 | 0.274 | 0.038 |
| **Infection-related biomarker** |  |  |  |  |  |
| C-reactive protein (ng/L, 0.0-8.0) | 46.54±29.42 | 63.37±26.65 | 17.63±21.30 | 0.000 | 0·000 |
| Erythrocyte sedimentation rate (mm/h, 0.0-20.0) | 58·13±30·98 | 90.42±31.07 | 64.27±35.53 | 0.070 | 0.000 |
| **Blood gas analysis** |  |  |  |  |  |
| PaO_2_ (mmHg, 80.0-100.0) | 77.78±25.08 | 55.49±13.30 | 81.69±15.45 | 0.430 | 0.000 |
| PaCO_2_ (mmHg, 35.0-45.0) | 33.64±5.44 | 45.29±9.65 | 38.21±6.84 | 0.000 | 0.000 |
| Lactic acid (mmol/L, 0.5-1.6) | 4.08±3.23 | 6.24±6.34 | 1.60±3.52 | 0.003 | 0.000 |
| PaO_2_/ FiO_2_ (mmHg) | 318.52±130.00 | 203.10±93.67 | 361.45±97.45 | 0.098 | 0.000 |
| **APACHE Ⅱ score** | 11.03±3.51 | 18.58±7.28 | 6.20±10.03 | 0.000 | 0.000 |

^*^P<0.05 is of statistical significance

^#^Comparison between on admission and on discharge

^&^Comparison between peak and on discharge

Table S3 Clinical outcomes of 40 severe cases with COVID-19

|  | Number (percentage) |
| --- | --- |
| Tendency of pulmonary radiograph abnormalities |  |
| Improving | 36 (90.0%) |
| Stable | 2 (5.0%) |
| Deteriorating | 2 (5.0%) |
| Complications |  |
| Acute renal dysfunction^#^ | 20 (50.0%) |
| Acute liver dysfunction^*^ | 16 (40.0%) |
| Secondary pulmonary infection | 6 (15.0%) |
| Acute myocardial injury^$^ | 4 (10.0%) |
| Shock | 3 (7.5%) |
| Acute respiratory distress syndrome | 3 (7.5%) |
| Multiple organ dysfunction syndrome | 4 (7.5%) |
| Disseminated intravascular coagulation | 2 (5.0%) |
| Gastrointestinal hemorrhage | 1 (2.5%) |
| Outcomes |  |
| Hospitalization | 0 (0.0%) |
| Discharge | 38 (95.0%) |
| Death | 2 (5.0%) |

^#^Alanine aminotransferase increased by ≥ 100% or development of jaundice.

^*^Elevated blood urea nitrogen or creatinine above the normal upper limits.

^$^Creatine kinase-MB activity increased by ≥ 100%.
